# The Time Is Right to Focus on Model Organism Metabolomes

**DOI:** 10.3390/metabo6010008

**Published:** 2016-02-15

**Authors:** Arthur S. Edison, Robert D. Hall, Christophe Junot, Peter D. Karp, Irwin J. Kurland, Robert Mistrik, Laura K. Reed, Kazuki Saito, Reza M. Salek, Christoph Steinbeck, Lloyd W. Sumner, Mark R. Viant

**Affiliations:** 1 Departments of Genetics and Biochemistry, Complex Carbohydrate Research Center and Institute of Bioinformatics, University of Georgia, 315 Riverbend Road, Athens, GA 30602, USA; aedison@uga.edu; 2 Wageningen University & Research Centre, PO Box 16, 6700AA Wageningen, The Netherlands; robert.hall@wur.nl; 3 CEA, iBiTec-S, Service de Pharmacologie et d’Immunoanalyse, Laboratoire d’Etude du Métabolisme des Médicaments, MetaboHUB-Paris, CEA Saclay, Building 136, 91191 Gif-sur-Yvette cedex, France; christophe.junot@cea.fr; 4 Bioinformatics Research Group, SRI International, 333 Ravenswood Avenue AE206, Menlo Park, CA 94025, USA; pkarp@ai.sri.com; 5 Albert Einstein College of Medicine, 1301 Morris Park Avenue, Bronx, NY 10461, USA; irwin.kurland@einstein.yu.edu; 6 HighChem LLC, Leskova 11, 81105 Bratislava, Slovakia; robert.mistrik@highchem.com; 7 Department of Biological Sciences, University of Alabama, 300 Hackberry Lane, Tuscaloosa, AL 35487, USA; lreed1@ua.edu; 8 RIKEN Center for Sustainable Resource Science, Yokohama 230-0045; Chiba University, Chiba 260-8675, Japan; kazuki.saito@riken.jp; 9 European Bioinformatics Institute (EMBL-EBI), European Molecular Biology Laboratory, Wellcome Trust Genome Campus, Hinxton, Cambridge, CB10 1SD, UK; reza.salek@ebi.ac.uk (R.M.S.); steinbeck@ebi.ac.uk (C.S.); 10 University of Missouri, Department of Biochemistry, Columbia, MO 65211, USA; sumnerlw@missouri.edu; 11 School of Biosciences, University of Birmingham, Birmingham B15 2TT, UK

**Keywords:** *Escherichia coli*, *Saccharomyces cerevisiae*, *Caenorhabditis elegans*, *Danio rerio*, *Daphnia magna*, *Drosophila melanogaster*, *Mus musculus*, *Arabidopsis thaliana*, *Oryza sativa*, *Solanum lycopersicum*

## Abstract

Model organisms are an essential component of biological and biomedical research that can be used to study specific biological processes. These organisms are in part selected for facile experimental study. However, just as importantly, intensive study of a small number of model organisms yields important synergies as discoveries in one area of science for a given organism shed light on biological processes in other areas, even for other organisms. Furthermore, the extensive knowledge bases compiled for each model organism enable systems-level understandings of these species, which enhance the overall biological and biomedical knowledge for all organisms, including humans. Building upon extensive genomics research, we argue that the time is now right to focus intensively on model organism metabolomes. We propose a grand challenge for metabolomics studies of model organisms: to identify and map all metabolites onto metabolic pathways, to develop quantitative metabolic models for model organisms, and to relate organism metabolic pathways within the context of evolutionary metabolomics, *i.e.*, phylometabolomics. These efforts should focus on a series of established model organisms in microbial, animal and plant research.

## 1. The Importance of Model Organisms

Model organisms are an essential component of biological, biomedical and environmental research that can be used to study specific biological processes. They represent carefully selected biosystems that are relatively simple to manipulate, are relatively inexpensive, and consequently are widely available and accessible to researchers. These biosystems often have short generation times and are easy to propagate. Model organisms can include vertebrate and invertebrate animals, plants and microbes. Intensive studies of a moderate number of key organisms yield research efficiencies, and is leading to systems-level understandings of these organisms. Furthermore, knowledge gleaned for model organisms can be transferred to related organisms. The most cited organisms within Google scholar comprise *Homo sapiens, Mus musculus, Arabidopsis thaliana, Escherichia coli, Saccharomyces cerevisiae, Caenorhabditis elegans* and *Drosophila melanogaster*. Five of these non-human species form a subset of the 13 model organisms listed by the US National Institutes of Health (NIH).

Much of what we know about biological processes, whether they be involved in human disease, pharmacology, toxicology, plant physiology, evolution, or ecology, has been learnt through basic research into model organisms. For example, major insights into developmental biology have been gained from research on fruit flies (in particular *D. melanogaster*). It is an ideal species for this purpose as it is easy to maintain, small, breeds rapidly, and lays many eggs. In addition, *D. melanogaster* has many characterized visible mutations, only four chromosomes and a well-annotated genome sequence. These resources have led to its rapid establishment as one of the most widely used model organisms with over 91,000 references catalogued in PubMed. *C. elegans*, a free-living transparent nematode, has also become an excellent model for developmental biology. Horvitz and Sulston followed the fate of all cell divisions from a fertilized egg to an adult hermaphrodite [1], enhancing our understanding of normal development and leading to the discovery of apoptosis. Increasingly this species is being used as a model organism in terrestrial toxicology. Another invertebrate, the water flea *Daphnia*, is the most recently listed NIH model organism. It has long been an established sentinel species in freshwater ecology and regulatory toxicology. *Daphnia’*s listing by the NIH followed from the discovery that its genome is highly responsive to environmental stresses [2], and hence can serve as a model for both human-environment interactions as well as in freshwater toxicology. Mouse models are of course widely used in biomedical research, with €550 M European Commission funding invested into research projects using this model between 1998 and 2010 [3]. Zebrafish (*Danio rerio*) represent another important vertebrate model that shares a high degree of genetic and organ system homology to humans. Advantages for the use of zebrafish, in comparison to mice, include the ability to house zebrafish in more natural conditions than is possible to simulate for mammals, ease of genetic manipulation (e.g., through absorption of chemical mutagens added to their water), much higher fecundity, rapid growth and development, and transparency through early adulthood allowing the use of imaging modalities for metabolic and phenotypic assessments. This species has wide-ranging applications as a model from biomedical to environmental research. Within the plant kingdom *A. thaliana*, a member of the mustard (*Brassicaceae*) family, is widely used as a model organism in plant biology, including plant-pathogen interactions, a topic of wide-ranging importance in times of concern about feeding a growing population on the planet.

While seemingly disparate, research into bacteria, yeast, insects, worms, fish, rodents and plants has shown that the core biochemical operating principles have been conserved across all living organisms. It is this very fact that justifies how findings derived from non-mammalian animals, for example, can shed light on biological processes in humans. For the specific case of metabolic biochemistry, primary metabolites have traditionally been defined are those directly involved in conserved processes necessary for life such as energy generation, growth, development and reproduction, and hence are typically present across many taxa. In contrast, secondary metabolites have traditionally been defined as not being directly involved in conserved processes and instead have a role(s) in more specialized metabolism. Secondary metabolites are vastly more chemically diverse and occur widely in plants, fungi and microbes and are of huge importance to many industries (e.g., the pharmaceutical, nutraceutical and agrochemical sectors). A systematic and comparative study of the metabolism of several model organisms will help us to better understand universally conserved, as well as more specialized pathways, which ultimately should lead to a deeper understanding of metabolites beyond their simple categorization into “primary” and “secondary”.

## 2. Growth of ’Omics Research into Model Organisms

The sequencing of organism genomes, exemplified by the Human Genome Project that was launched in 1990, represents one of the greatest endeavors in the history of science. This project and associated technologies helped to drive the sequencing of many other organisms and consequently the continued growth of model organism research, with scientific communities further expanding around key species as their genomes were sequenced. For example, considering the model organisms introduced above, *A. thaliana* was the first plant genome to be sequenced in 2000 [4] and there is now a vast array of resources and many are outlined in the Arabidopsis Information Resource [5]. The draft mouse genome was published shortly after the human genome in 2002 [6]. The sequencing of *Daphnia pulex* was the first of any crustacean, revealing that it contained many more genes than the human genome [2]. However, these activities have not solely been driven by the sequencing of the human genome. As noted by Wilson and others, the *C. elegans* genome-sequencing project provided an important foundation upon which to develop the Human Genome Project [7]. Furthermore, the ENCODE project sponsored by National Human Genome Research Institute (NHGRI) [8] to accelerate the annotation of functional non-coding portions of the human genome was facilitated and complemented by the modENCODE project that focused on similar genomic regions in *C. elegans* and *D. melanogaster* [9]. By comparison to the human reference genome, approximately 70% of human genes have at least one zebrafish ortholog [10]. The importance of these model organisms, and the growth of genomics research focused on these species, cannot therefore be overstated.

Beyond genomics, the Human Proteome Organization (HUPO) has launched an initiative on Model Organism Proteomes (iMOP). The overarching aim of iMOP is to create a global network of experimental and bioinformatics groups interested in model organism proteomes, to standardize the protocols and standards for model organism proteome characterization, and to make optimum use of the proteomics data once acquired [11]. Collectively, these large-scale data generation experiments have fed the development of new computational techniques for genome-scale metabolic modeling. From annotated genomes we can now computationally infer the reaction network of an organism [11], which in turn can be used to develop a steady-state metabolic model for that organism [12]. Genome-scale metabolic models have now been developed for the model organisms *E. coli* [13,14] and *S. cerevisiae* [15], as well as for humans [16]. These models can accurately predict gene knock-out phenotypes, growth rates under different nutrient conditions, and disease phenotypes.

The field of metabolomics has truly come of age and is making a significant impact in a wide range of fields from biomedicine and pharmacology to agriculture [17,18,19,20]. Several metabolomics studies have revealed an unexpectedly large uncharted landscape of unknown metabolites in the organisms studied to date [21,22,23]. These metabolites, and the biochemical pathways that produce them, represent a huge opportunity for new biological discovery. It was in response to this challenge that the international Metabolomics Society launched a Metabolite Identification task group in 2013 [24], with goals to develop robust reporting standards for metabolite identification and to advertise best-practice to the metabolomics community using a range of analytical approaches [25,26,27,28]. The challenge of identifying all metabolites in all sample types is currently—and into the foreseeable future—not achievable. This paucity of peak identification is often, and justifiably, highlighted as one of the critical weaknesses of metabolomics science by those outside of the metabolomics community. Hence it is vital that this challenge is confronted immediately, drawing upon broad international expertise.

## 3. Launch of the Model Organism Metabolomes Task Group

To leverage both the international studies of model organisms and the rapidly growing field of metabolomics, as well as to add momentum to more general efforts to identify metabolites, we propose that the community should focus and extend research activities specifically on the identification of Model Organism Metabolomes (MOMs). In June 2015, the authors of this paper met in San Francisco and launched the Metabolomics Society’s Model Organism Metabolomes (MOM) task group, and have subsequently developed a grand challenge as well as multiple aims for the metabolomics and related communities. The grand challenge is to *identify and map all metabolites onto metabolic pathways, to develop quantitative metabolic models for many model organisms (Table 1), and to relate organism metabolic pathways within the context of evolutionary metabolomics, i.e., phylometabolomics*. More specifically, we define *phylometabolomics* as the comparative analysis of the evolution of metabolism and metabolic networks in a phylogenetic context. Such an approach is dependent upon our ability to identify the metabolomes of multiple species and would enable us to study, for example, the roles of ancestral constraint, common ancestry and the evolutionary convergence of metabolism. In turn, such knowledge has practical applications, for example to predict human or environmental toxicity through the discovery of evolutionarily conserved molecular mechanisms of toxic responses, or to highlight metabolic pathway characteristics of species longevity [29]. We recommend that these efforts to identify and map all metabolic pathways should focus initially on a series of established model organisms in microbial, animal and plant research (Table 1). Furthermore, these efforts will exploit established tools from all of the model organisms to provide novel biological and bioinformatic approaches for metabolite identification through genetic manipulations, including gene knockouts, transgenics, mutations, and large panels of recombinant inbred lines. These genetic manipulations are not ethically possible in humans but will provide information that can be applied to humans and human disease.

**Table 1:** Prioritized list of model organisms that the new MOM task group recommend for deep investigations of their metabolomes.

Kingdom	Latin Name	Common Name
Bacteria	*Escherichia coli*	-
Fungi	*Saccharomyces cerevisiae*	yeast
Animal (invertebrate)	*Caenorhabditis elegans*	nematode
	*Daphnia magna*	water flea
	*Drosophila melanogaster* *	fruit fly
Animal (vertebrate)	*Danio rerio*	zebrafish
	*Mus musculus*	mouse
Plant	*Arabidopsis thaliana* **	thale cress
	*Medicago truncatula*	barrel medic, model legume
	*Oryza sativa*	rice
	*Solanum lycopersicum*	tomato

* International Drosophila Metabolomics Curation Consortium [30]; ** Metabolomics subcommittee (chaired by Kazuki Saito) within the Multinational Arabidopsis Steering Committee [31].

The aims of the Model Organism Metabolomes task group are:

- To integrate disparate model organism-focused research groups into a model organism metabolomes community, to promote interactions between these groups, and to stimulate joint initiatives.
- To share, discuss and coordinate analytical strategies to progress the annotation and identification of model organism metabolomes (including polar metabolites and lipids), linking with the efforts of the Metabolomics Society’s Metabolite Identification task group, to generate best-practice strategies. In addition, to determine quantifiable ranges of primary and secondary metabolite levels measured in the model organisms under differing conditions, e.g., diet and environment.
- To share, discuss and coordinate bioinformatic strategies, data standards and databases for the curation, analysis and visualization of model organism metabolomes, to generate/best-practice strategies.
- To catalyse the integration of metabolome data with the huge battery of existing information and knowledge of model organisms, thereby linking metabolic data with other data types including other ’omics data.
- To catalyze the integration of phylometabolomic data into a framework for understanding the evolution of metabolic networks and also how these respond to stressors, and to explore linkages between the phenotype(s) induced by these stressors.
- To support the development of quantitative metabolic models for model organisms.
- To promote the use of model organism metabolomes in systems biology education for training future ’omics scientists.

Meetings that include a focus on model organism metabolome identification include both past events (e.g., Genetics Society of America *Drosophila* conference 2014, Metabolomics Society conference 2015) as well as proposed future events (e.g., the Metabolomics Society’s 2016 conference and beyond, Daphnia Genomics Consortium conferences, GSA Model Organism conferences). To initiate more immediate discussions within the community, the MOM task group has established a forum at http://metabolomics-forum.com. In addition, we recommend either the MetaboLights repository [32] based at EMBL-EBI [33] or Metabolomics Workbench [34,35] for data deposition related to model organism species. Furthermore, we recommend resources such as the BioCyc and Pathways Tools to build curated predicted metabolic networks from annotated genome sequences for model organisms [36,37]. We welcome the opportunity to dialogue with the international metabolomics community, to discuss how to address the aims above, and together to advance the annotation and identification of model organism metabolomes.
